# Investigating the Interaction Between Prosody and Pragmatics Quantitatively: A Case Study of the Chinese Discourse Marker *ni zhidao* (“You Know”)

**DOI:** 10.3389/fpsyg.2021.716791

**Published:** 2021-12-06

**Authors:** Yi Shan

**Affiliations:** ^1^School of Foreign Studies, Nantong University, Nantong, China; ^2^College of Foreign Languages, Fujian Normal University, Fuzhou, China; ^3^School of Foreign Languages, Nanchang Institute of Technology, Nanchang, China

**Keywords:** discourse marker, prosody, pragmatic function, interaction, prosody-pragmatics model

## Abstract

This study briefly describes the prosodic and pragmatic characteristics of the discourse marker *ni zhidao* (“you know”) in spoken Chinese. It mainly explores the interaction between its prosody and pragmatics using instrumental methods. It is the first attempt to use acoustic and statistical analysis to examine the prosodic parameters and prosody-pragmatics interaction of a Chinese discourse marker. The corpus includes 71 interview conversations totaling more than 30 h, in which 490 discourse marker tokens of *ni zhidao* were found. *Ni zhidao* mainly fulfilled four broad pragmatic functions of initiating a topic when occurring sentence-initially, of holding the floor when appearing within clauses, of marking coherence when making its presence between clauses, and of projecting attitudes and feelings when showing up sentence-finally. Drawing on the algorithm of random forest in R, the acoustic and statistical analysis of the performance of *ni zhidao* in these four functions showed that its prosodic features, including duration, tempo, pre-pause, post-pause, F_0_, and intensity, significantly relate to and thus imply its pragmatic functions, that the interaction between its prosody and pragmatics can be modeled statistically, and that the established pragmatics classification model based on prosody can be utilized to predict the pragmatics of *ni zhidao*. These findings seem to strengthen the hypothesis that prosodic variables play a role in deciphering the different pragmatic functions of *ni zhidao*. This study uses prosodic evidence to more objectively reveal not only the part of *ni zhidao* in dynamically constructing and embodying specific contexts but also its communicative functions and the underlying meta-pragmatic awareness behind it. This study breaks through the limitations of traditional discourse marker research, which mainly relies on context and discourse characteristics for subjective reasoning.

## Introduction

In essence, discourse markers (e.g., *ni zhidao*, *you know*, etc.) are linguistic items to guide the communicator’s understanding of the discourse during the communicative process. They both indicate the purpose of the speaker’s discourse accurately and guide the listener to understand it, thus effectively realizing the communicative intent. Related studies (e.g., Blakemore, 1987; Fraser, 1987; Schiffrin, 1987; Chen, 2002; He and Mo, 2002; Ran, 2002; Fang, 2005; Wu, 2005; Tanno, 2018; Rhee, 2020) have analyzed the syntactic distribution, communicative process, causes of formation, and development process of discourse markers. These researches reveal their syntactic features, discourse functions, grammaticalization, and pragmaticalization, thus deepening the academic understanding of this pragmatic phenomenon.

The pragmatic functions of discourse markers have traditionally been the focus of scholarly attention. To date, researchers (e.g., Blakemore, 1987; Fraser, 1987; Schiffrin, 1987; Chen, 2002; He and Mo, 2002; Ran, 2002; Fang, 2005; Wu, 2005; Tanno, 2018; Rhee, 2020) have primarily relied on context and syntax to examine the multiple pragmatic functions of specific discourse markers in different contexts, or to analyze how discourse markers perform certain discourse functions in specific contexts within some theoretical frameworks. These investigations need to be further developed and refined for four reasons: (1) over-relying on abstract communicative contexts for subjective reasoning; (2) lacking sufficient linguistic evidence due to investigation into only a small number of examples; (3) failing to statistically investigate the interaction between “tangible” prosody and “intangible” function of discourse markers; (4) mainly using elicited conversations as data and paying little or even no attention to spontaneous speech.

Although context can be used to identify the pragmatic functions of discourse markers, prosody can undoubtedly provide another objective and easily accessible evidence to facilitate listeners’ understanding. The crucial functional load of semantically unspecified discourse markers is carried by prosodic variation (Gravano et al., 2007; Lai, 2009). However, the prosodic realization of discourse markers has received little attention (Wichmann et al., 2010) because they are more typical of spoken than written language (Brinton, 1996). Although claiming that the prosody of discourse markers performs some pragmatic functions, studies in this respect (e.g., Schegloff, 1982; Ward and Tsukahara, 2000; Ward, 2006; Gravano and Hirschberg, 2009; Buschmeier et al., 2011; Nadeu and Prieto, 2011; Nebot, 2021) fail to substantiate this prosody-pragmatics interaction with quantitative, acoustic-prosodic evidence. Realizing this neglect and failure, some linguists (e.g., Matzen, 2004; Braga and Marques, 2004; Wichmann et al., 2010; Volín et al., 2016; Didirková et al., 2018) have investigated what an analysis of prosody can reveal about the pragmatics of discourse markers. However, they neither investigated the prosody-pragmatics interaction of discourse markers statistically nor modeled this interaction and then used the model to predict pragmatic functions.

Given the current state of research, this study aims to statistically examine and model the interaction between the prosody and pragmatics of the discourse marker *ni zhidao* in Chinese spontaneous speech based on a corpus of media interview conversations totaling more than 30 h. To this end, six prosodic parameters, including duration, tempo, pre-pause, post-pause, F_0_, and intensity, were chosen, and the pragmatic functions of *ni zhidao* were impressionistically identified through careful listening to the audios. The hypothesis underlying this study is that prosodic variables play a role in encoding and deciphering different pragmatic functions of *ni zhidao* in diverse utterance positions. This hypothesis draws on the literature concerning the correlation between prosody and position on the one hand and the interaction between prosody and pragmatics on the other hand. The existent researches (e.g., Vaissière, 1983; Cai et al., 1998; Xu, 1999; Zhong et al., 2001; Hirschberg, 2002; Wu, 2002; Ward, 2004; Braga and Marques, 2004; Wang, 2011) confirmed the impact of position on F_0_. The discourse component at the beginning of the turn is higher in pitch, which is usually used by the speaker to take over the turn (Braga and Marques, 2004), because the speaker draws the listener’s attention to the subsequent discourse by raising the pitch (Ward, 2004). The pitch of the phrase initiating a new topic is higher than that of other components in the same turn (Hirschberg, 2002). The phrase at the end of the sentence has a narrower F_0_ range, a slower pitch, and a faster tempo (Hirschberg, 2002), which is the final lowering effect, that is, the sentence-final phrase carries the lowest pitch (Wang, 2011), due to compression of the pitch range during the last half-second or so of an utterance (Caspers, 1998). The aforementioned literature justifies the possibility that prosodic cues of *ni zhidao* could be predicted by its positions. “Different positions are responsible for subtle changes in meaning or function.” (Hansen, 1997: 156) Therefore, it is reasonable to hypothesize that the prosodic cues of *ni zhidao* can reliably be used to classify its pragmatic functions. As has been proved in previous studies, systematic prosodic variation is functionally extrinsically motivated (Volín et al., 2016). Prosodic patterns are basically composed of different functional layers (Xu, 2004). The crucial functional load of discourse markers is carried by their varying prosodic patterns (Gravano et al., 2007; Lai, 2009). Prosodic parameters interact intricately to convey various communicative functions, and multi-parametric variations in F_0_, timing, and intensity result in basically consistent form-function mappings (Volín et al., 2016). The prosodic elements are integrated to produce tailored discourse markers serving specifically intended communicative functions (Ward, 2006). Therefore, it is well-grounded and feasible to use prosodic clues of *ni zhiao* as solid evidence for its pragmatic categorization.

It is the first attempt to use acoustic and statistical analysis to probe the prosodic parameters and prosody-pragmatics interaction of Chinese discourse markers. Specifically, the present research analyzed the syntactic, prosodic, and pragmatic distribution of *ni zhidao*, the correlation between its prosodic variables, and ultimately its prosody-pragmatics correlation through the machine learning algorithm (Random Forest in R). The syntactic, prosodic, and pragmatic analyses were designed to reveal its actual syntactic and prosodic performances statistically and its pragmatic functions intuitively for investigating the potential correlation between its prosody and pragmatics in different positions eventually. On the basis of these statistical and impressionistic analyses, the Random Forest algorithm was meant to project variations in prosody onto functional categories to find the significance of prosody to encoding and deciphering pragmatics statistically and construct a prosody-pragmatics model which can be applied to predict pragmatics, thus providing statistical evidence for the traditionally intuitively-claimed pragmatic functions and prosody-pragmatics interaction. As such, the investigation of *ni zhidao* first explored the prosody-pragmatics interface of Chinese discourse markers statistically and visually, using “visible” prosodic evidence rather than “invisible” context to more objectively reveal not only the part of *ni zhidao* in dynamically constructing and embodying specific contexts but also its communicative functions and underlying meta-pragmatic awareness. This study, therefore, breaks through the limitations of traditional discourse marker research, which primarily relied on context and discourse characteristics for subjective reasoning, putting discourse marker studies on a more objective and scientific footing.

## Review of Literature

### Studies of Discourse Markers

Research on discourse markers generally presents five representative perspectives: (1) Coherence model (e.g., Schiffrin, 1987, 1994, 2003; Chen, 2002; He and Mo, 2002) explores the role of discourse markers in displaying the semantic coherence between discourse segments and the discourse coherence mode; (2) Relevance model (e.g., Blakemore, 1987, 2002, 2011; Ran, 2002; Shan, 2014a; Li et al., 2018) focuses on relevant inference, studying how speakers use discourse markers to guide or restrict listeners to find relevance between discourse segments; (3) Syntactic-pragmatic model (e.g., Fraser, 1987, 1999, 2009, 2015; Akar and Öztürk, 2020) examines the syntactic features and pragmatic functions of discourse markers, arguing that the function of discourse markers is to guide the listener to correctly interpret the logical relationship between the preceding and following discourse segments; (4) Grammaticalization/pragmaticalization model (e.g., Fang, 2005; Wu, 2005; Maschler, 2009; Dong, 2010; Li, 2014; Fedriani and Sanso, 2017; Tanno, 2018; Rhee, 2020) probes into the evolution of discourse markers and the contributing factors behind this; (5) Prosody-pragmatics model (e.g., Hirschberg, 2002; Matzen, 2004; Braga and Marques, 2004; Wichmann et al., 2010; Beňuš, 2012; Abuczki, 2014; Cabarrão et al., 2015; Gonen et al., 2015; Volín et al., 2016) draws on prosody as an objective factor to identify and characterize discourse markers or as an immediate and readily accessible feature to reveal the functions of discourse markers and how people comprehend them. Some of these studies have shifted from the traditional syntactic-semantic perspective to the pragmatic-cognitive or even prosodic dimension. Some researchers (e.g., Wu, 2005; Dong, 2010; Li, 2014; Wang, 2017) have investigated Chinese discourse markers.

*Ni zhidao* among other Chinese discourse markers has relatively been under-explored, relative to discourse markers in other languages. Representative studies investigated the distinctive morphological features and attention-arousing and communication-checking functions of *ni zhidao* (Tao, 2003), attention-focusing, background-providing, and identification-seeking modes of *ni zhidao* (Liu, 2006), cognitive context-constructing, attitude-projecting, and inference-manifesting functions of *ni zhidao* (Shan, 2014a), discourse-constructing functions of *ni zhidao* and the internal mechanism and external motivation for its evolution (Shan, 2014b), and the prosody of *ni zhiao* in different positions (Shan, 2015). These researches neither made a statistical analysis nor constructed a model by integrating position, pragmatics, and prosody. In the same vein, among studies of discourse markers in other languages, some (e.g., Blakemore, 1987; Fraser, 1987; Schiffrin, 1987; Tanno, 2018; Rhee, 2020) examined the pragmatic functions of specific discourse markers relying on context and syntax, and some mainly focused on the multifunctionality of discourse markers (e.g., Schiffrin, 1987; Brinton, 1996; Jucker and Ziv, 1998; Lenk, 1998; Erman, 2001; Aijmer, 2002) and/or their syntactic positions (e.g., Fraser, 1990; Redeker, 1991; Hansen, 1997; Schourup, 1999; Schiffrin, 2001; Halliday and Matthiessen, 2004), without mapping pragmatic functions onto syntactic positions statistically. In this research state, a statistical analysis of the utterance distributions and pragmatic roles of *ni zhidao* and the mapping of the former onto the latter in particular can provide new insights into the intuitively-inferred correlation between the position and pragmatics of discourse markers that “different positions are responsible for subtle changes in meaning or function” (Hansen, 1997: 156). Another factor making the study of *ni zhidao* unique is that Chinese belongs to a language family different from the language families of most languages investigated in the literature. Thus, more cross-linguistic comparisons will be possible and the questions of universality of some characteristics will be addressed (Volín et al., 2016).

The phonological features on discourse markers mainly discussed in the literature are pauses, phonological reduction (Schiffrin, 1987; Brinton, 1996; Tabor and Traugott, 1998), and intonation (Hirschberg and Litman, 1987, 1993; Romero-Trillo, 2015). A pause before the discourse marker is usually expected if the discourse marker occurs initially in an intonation unit, and a pause after the discourse marker is frequent, and it is hinted at by the syntactic detachment (Schiffrin, 1987) of the discourse marker and by the “comma intonation” (Tabor and Traugott, 1998: 254). The pauses on both sides of the discourse marker form a separate intonation unit, or “an independent breath unit carrying a special intonation and stress pattern” (Traugott, 1995: 60). Hirschberg and Litman (1993) found that the English *well* proved to be prosodically independent in only 50% of cases. Der and Marko (2010) discovered that a discourse marker is not necessarily preceded and, or followed by a pause.

Prosodic features, including accent, intonation, tone, and pauses, are significant to realizing the speaker’s communicative intent (Searle, 1969). An intimate relationship exists between prosody and pragmatics (Ward, 2004). Turner (2002) classified the discourse marker *you know* in his corpus data into nine prosodic variants and studied their functions and distribution in various positions. Rendle-Short (2003) revealed the way the discourse marker *so* occurs in specific contexts with specific prosodic features and functions according to its position in the seminar. Braga and Marques (2004) identified some standard pragmatic functions for syllabification, duration, loudness, pitch height, pitch slope, and creaky voice in non-lexical sentences. He concluded that each of these prosodic features bears a reasonably consistent core meaning. Petukhova and Bunt (2009) have studied prosodic features such as pitch, energy, voicing, speaking rate, and segment duration. Matzen (2004) provided a descriptive analysis of the relationships between prosody and function for a discourse marker *so.* The results show that prosodic features distinguish the functional categories of *so* and that prosodic features can distinguish multifunctional tokens of *so* from those performing only one function. The conclusion is that prosodic features, in combination with context, are beneficial for elucidating the structure and usage of *so*. Wichmann et al. (2010) described the functions and prosodic realizations of *of course* in present-day spoken British-English and explored the relationship between prosody and grammaticalization. Their findings relate to structure, meaning and use, and prosody. In respect of construction, there is a clear preference for *of course* to occur in the initial position as part of the thematic material, followed by a medial position as a post-thematic marker to highlight the theme. In semantic terms, there is strong evidence of grammaticalization with more literal meanings occurring alongside subjective and inter-subjective development. It was predicted that semantic change involving a loss of semantic weight favoring pragmatic meaning would also include a loss of prosodic prominence. “Prosodic choices–segmentation, accent placement, and tone choice–convey abstract meanings that can be related only indirectly to lexical items and are motivated in part by convention, but largely by the often conflicting demands and constraints of the semantic, pragmatic and discoursal functions that discourse markers fulfill” (Wichmann et al., 2010:47). Didirková et al. (2018) found that the silent pause duration before the discourse marker, as well as the whole duration of the discourse marker itself, were used by the speaker to differentiate between the core meaning of the discourse marker and its less predictable meanings. Moreover, prosodic cues were not used redundantly, and the discourse markers did not systematically constitute a separate prosodic unit.

Much of the research on discourse markers, especially in computational linguistics, is concerned with the possible cues for disambiguation. Hirschberg and Litman (1987, 1993) studied *now*, aiming to identify its use as a “cue phrase” intonationally. According to them, prosody is the only feature that provides adequate information to distinguish between *now* as a cue phrase and a non-cue *now*. In the case of *like* and *well*, position in the sentence and the presence of a pause before or after the words in question were found helpful for the identification of discourse markers (Popescu-Belis and Zufferey, 2011). Other prosodic features have been studied in this direction too. Matzen (2004) found clear connections between the functions and prosody of *so*, considering length, pitch contour, sound, and position in the sentence. Stress was shown to be a diagnostic for discourse markers by Dehé and Wichmann (2010a,b). They found that the different functions of *I think* and *I believe* were distinguished by accent placement, while as discourse markers, these phrases were unstressed. The duration of the *like* in its various functions was explored by Gray (2010). Der and Marko (2010) attempted to identify the positional and/or acoustic properties aiding the listener in perceiving the function of a word. Beňuš (2012) looked at the relationship between the prosody and discourse/pragmatic meanings of Slovak feedback vocalization corresponding to the word *no*. The finding is that the function of back-channel/continuer is the most easily disambiguated by the pitch contour, duration, and other features. In contrast, other functions require more sophisticated multi-factor analyses for identifying the best disambiguating features. Van Zyl and Hanekom (2012) discovered that *okay* with a neutral agreement differs in prosody from *okay* with a reluctant agreement in the same discourse position. Gonen et al. (2015) and Hirschberg and Litman (1987, 1993) found that prosodic information facilitates the hearer distinguishing a discourse marker from its literal counterpart.

Some linguists have also studied non-English discourse markers. Abuczki (2014) found that the duration of the discourse marker was one of the two defining features distinguishing the different functions of the Hungarian discourse markers *mondjuk* (“let’s say”), *ugye* (“is that so?”), and *amúgy* (“otherwise”). Cabarrão et al. (2015) used prosodic features to categorize discourse markers in two speech corpora of European Portuguese. Gonen et al. (2015) described the discursive characteristics of the discourse marker *axshav* (“now”) in spoken Hebrew and explored its prosodic features. The finding is that most discourse markers had characteristic intonation contours, including a sharp decrease in the frequency inside the second syllable. It was also discovered that the duration of the performance of *axshav* as a discourse marker was shorter, both for the performance of the first syllable and the overall duration of the word, compared with its performance as a temporal adverbial. Volín et al. (2016) examined the prosodic forms that expressed eight pragmatic functions of the Czech discourse marker *jasnì* [“sure,” “agreed,” “of course,” “okay,” “(al)right” or “fair enough”], including resignation, reassurance, surprise, indifference, or impatience. They proposed multi-parametric differences between *jasnì* realizations in terms of their F_0_, timing, and intensity patterns, which gave rise to generally consistent form-function mappings.

As is shown in the literature above, the prosodic investigations of discourse markers either only scrutinized some prosodic variables, like accent, intonation, tone, and pauses, or merely examined certain parameters, including F0, duration, and intensity, which were used to distinguish communicative functions or disambiguate between discourse markers and their literal counterparts. However, these researches did not map prosody onto pragmatics statistically. Recent studies tried to fill this gap. The most representative, Volín et al. (2016), made a step further, exploring the form-function mappings through statistics and modeling, but they only probed a turn-initial discourse marker. The current study of *ni zhidao* attempts to go even further in this direction by integrating six overriding prosodic parameters into the examination of four functions of *ni zhidao* in four utterance positions. Such an analysis is likely to shed new light on future discourse marker studies not only in terms of a panoramic view but also with regard to cross-linguistic evidence for linguistic peculiarity and universality, thus allowing to “further simplify the complex form-function links in discourse markers” (Volín et al., 2016).

### Studies of Prosody

As one of the intrinsic properties of discourse, prosody usually refers to three speech characteristics, including pitch, duration, and intensity (Wang, 2011). It constructs the context in which discourse intentions are interpreted, having a significant impact on the construction of discourse meaning and the interpretation of discourse functions. Studies in this respect (e.g., Li, 2002; Lin, 2002; Wu, 2002; Cao, 2003; Local and Walker, 2004; Dehé and Wichmann, 2010a,b; Sohn and Kim, 2014; Ma, 2017) have mainly explored the phonological characteristics of accent, intonation units, prosodic words, prosodic facets, and prosodic chunks based on small-sized corpora of daily speech. Only a few studies (e.g., Hirschberg and Swerts, 1998; Terken and Swerts, 2002; Xiong, 2003; Xiong and Lin, 2004) have examined the relationship between prosody and pragmatics. These studies argue that the prosodic features of discourse are primarily constrained by its communicative functions. The communicative functions of speech are, to some extent, achieved through the prosodic features.

Lucien Brown is a crucial player in prosody research successfully incorporating mainstream linguistic research on prosody into present-day pragmatics. According to Winter et al. (2013), politeness is not only expressed by honorific lexical forms commonly employed in Korean but also by speech acoustics. Brown et al. (2014) found that politeness does not merely reside in verbal markers but is co-signaled by phonetic cues. Brown and Prieto (2017) looked at how (im)politeness is communicated through prosody. They found that multiple acoustic features pattern with politeness-and impoliteness-related meanings, including fundamental frequency (pitch), duration (length), intensity (loudness), and various aspects of voice quality, including breathiness.

These studies of prosody reveal the tone and mood of the speaker with sound waves, speech spectrum, pitch and intensity lines, and the corresponding data. They reflect the mechanisms and intentions underlying the discourse, but they fail to probe the prosody of discourse markers. The speaker’s tone, attitude, and emotions can change the prosodic characteristics of discourse markers, and this change precisely reflects the speaker’s mind and psychology. Discourse markers, as essential components of meta-language, are more reflective of the speaker’s meta-pragmatic awareness compared with other discourse components. Examining the prosody of discourse markers can essentially reveal the discourse motivation behind the choice of discourse components. Therefore, the prosody of discourse markers should be incorporated into the study of prosody as a whole.

### Corpus-Based Computational Studies of the Usage of Language

Many empirical studies of language usage based on large-scale authentic corpora have been conducted across the world. These studies calculate and display information on language usages, such as lexical collocation and semantics (e.g., Gao and Wei, 2017; Gries, 2017), semantic prosody (e.g., Fujisaki and Sudo, 1971; Wei, 2006), syntax (e.g., Wang, 2003), literary texts (e.g., Fang, 2016; Liu and Wang, 2017), translation styles (e.g., Liao, 2000; Hu and Xie, 2017), academic language (e.g., Hyland and Tse, 2012; Wang and Liu, 2017; Wei, 2017), and language teaching (e.g., Wang and Zhu, 2005; He, 2010; Chen and Liang, 2017). These typical case studies reveal the internal mechanisms for, and external influences on, certain language features. The results uncover some actual language usages and the meanings, functions, and thoughts conveyed by them. Based on sufficient evidence, these results of empirical studies are sound and convincing.

Statistical analyses based on corpora represented essential breakthroughs in research methodology. Such analyses have also updated the frameworks for language description and the views on language. They have put empirical studies of language usages on the footing of combining quantitative, qualitative, and interpretative approaches. However, none of the existing statistical analyses have focused on discourse markers and their prosodic features. Prosody as an essential part of speech (Wang, 2011) and discourse markers as distinctive features of natural discourse should carry weights in the empirical studies of language usages. Phonetic techniques and computational statistics can analyze the prosody and pragmatics of discourse markers in the form of spectrograms, statistical reports, and models. Therefore, the abstract and elusive meta-pragmatic awareness can be visualized to a large extent, and the rationale for using discourse markers can be revealed more objectively.

However, “finding statistical models appropriate for dealing with the complexities of human speech is an ongoing challenge for the field of linguistics,” and “this is a much-needed area of future research, which in turn would be highly beneficial to the investigations of the function and prosody of discourse markers” (Matzen, 2004).

## Experiments and Methods

The existing leading Chinese language corpora, including the Modern Chinese Corpus designed by the China National Language Commission, the Modern Chinese Corpus established by Peking University, the BCC Chinese Corpus of Beijing Language and Culture University, and the Media Language Corpus of China Media University, do not contain speech corpora. The Mandarin Speech Corpus for Four Major Dialects and the Media Speech Corpus are designed to fill this gap, but these two audio corpora are still under construction and cannot be used for this study. Drawing on the construction methods for these two corpora, the present study drew on ten representative media interview programs, such as PEOPLE IN THE NEWS, KE FAN QING TING, FEI CHANG DAO, MING REN MIAN DUI MIAN, QIANG QIANG SAN REN XING, etc., to create an audio corpus^1^ of more than 32 h. This corpus involves 71 interview conversations and 102 speakers, consisting of 12 interviewers and 90 interviewees. An overall of 536 tokens of *ni zhidao* was found, among which 490 discourse marker tokens (91%) and 46 non-discourse-marker tokens (9%) were identified by the author and verified by an expert team of five doctors majoring in syntax and five doctors majoring in pragmatics, based on the defining characteristics of discourse markers proposed by Schiffrin (1987: 328). Of the 490 discourse marker tokens, 91 (18.6%) were uttered by 10 interviewers, and 399 (81.4%) were produced by 73 interviewees. The profile of the corpus can be shown in Tables 1, 2.

**TABLE 1:** Profile of the interview programs.

Media channels	Programs	Number of Host (s)	Number of Guests
CCTV-1	XIAO CUI SHUO SHI	2	16
	PEOPLE IN THE NEWS	1	4
Dragon TV	KE FAN QING TING	1	9
	YANG LAN ONE ON ONE	1	5
Hunan Satellite Television	HER VILLAGE	2	10
Phoenix Satellite Television	LU YU YOU YUE	1	9
	MING REN MIAN DUI MIAN	1	9
	QIANG QIANG SAN REN XING	1	11
	FEI CHANG DAO	1	4
MSN	XING YUE DUI HUA	1	13
Total	10	12	90

**TABLE 2:** Profile of the audio data of 71 interview conversations.

Programs	File name	Duration	Overall *ni zhidao* tokens	Tokens by interviewer	Tokens by interviewee
XIAO CUI SHUO SHI	Wav.1	9′02″	2	2 by interviewer 1 0 by interviewer 2	0 by interviewee 1
	Wav.2	14′55″	2	0 by interviewer 1 0 by interviewer 2	0 by interviewee 2
	Wav.3	17′48″	6	0 by interviewer 1 0 by interviewer 2	2 by interviewee 3 2 by interviewee 4
	Wav.4	21′55″	6	3 by interviewer 1 3 by interviewer 2	0 by interviewee 5
	Wav.5	22′54″	4	0 by interviewer 1 0 by interviewer 2	2 by interviewee 6 2 by interviewee 7
	Wav.6	23′30″	6	6 by interviewer 1 0 by interviewer 2	0 by interviewee 8
	Wav.7	11′57″	5	2 by interviewer 1 0 by interviewer 2	3 by interviewee 9
	Wav.8	18′32″	10	0 by interviewer 1 0 by interviewer 2	8 by interviewee 10 2 by interviewee 11
	Wav.9	23′12″	7	3 by interviewer 1 0 by interviewer 2	4 by interviewee 12
	Wav.10	22′44″	12	0 by interviewer 1 0 by interviewer 2	12 by interviewee 13
	Wav.11	18′35″	4	0 by interviewer 1 2 by interviewer 2	2 by interviewee 14
	Wav.12	19′56″	4	4 by interviewer 1 0 by interviewer 2	0 by interviewee 15
PEOPLE IN THE NEWS	Wav.18	20′39″	8	2 by interviewer 3	6 by interviewee 16 0 by interviewee 17 0 by interviewee 18
YANG LAN ONE ON ONE	Wav.13	10′44″	3	3 by interviewer 4	0 by interviewee 19
	Wav.14	24′29″	10	2 by interviewer 4	8 by interviewee 20
	Wav.15	22′21″	9	3 by interviewer 4	6 by interviewee 21
	Wav.16	23′48″	11	4 by interviewer 4	7 by interviewee 22
	Wav.17	10′50″	3	0 by interviewer 4	3 by interviewee 23
KE FAN QING TING	Wav.19	20′57″	8	0 by interviewer 5	8 by interviewee 24
	Wav.20	12′53″	5	0 by interviewer 5	5 by interviewee 25
	Wav.21	12′17″	6	0 by interviewer 5	6 by interviewee 26
	Wav.22	13′59″	6	0 by interviewer 5	6 by interviewee 27
	Wav.23	5′46″	5	2 by interviewer 5	3 by interviewee 28 0 by interviewee 29
	Wav.24	18′23″	7	0 by interviewer 5	7 by interviewee 30
	Wav.25	27′52″	9	0 by interviewer 5	9 by interviewee 31
FEI CHANG DAO	Wav.26	20′23″	10	0 by interviewer 6	10 by interviewee 32
	Wav.27	17′52″	6	0 by interviewer 6	6 by interviewee 33
	Wav.28	1 h 8′11″	32	0 by interviewer 6	32 by interviewee 34
LU YU YOU YUE	Wav.29	21′20″	7	0 by interviewer 7	7 by interviewee 35
	Wav.30	21′23″	6	0 by interviewer 7	6 by interviewee 36
	Wav.31	9′15″	4	0 by interviewer 7	4 by interviewee 37
	Wav.32	31′10″	4	0 by interviewer 7	4 by interviewee 38
	Wav.33	29′07″	9	0 by interviewer 7	9 by interviewee 39
	Wav.34	32′07″	10	0 by interviewer 7	10 by interviewee 40
	Wav.35	1 h 35′18″	38	2 by interviewer 7	36 by interviewee 41
QIANG QIANG SAN REN XING	Wav.36	21′23″	6	0 by interviewer 8	6 by interviewee 42 0 by interviewee 43
	Wav.37	21′22″	5	0 by interviewer 8	5 by interviewee 44 0 by interviewee 45
	Wav.38	21′08″	6	4 by interviewer 8	2 by interviewee 46 0 by interviewee 47
	Wav.39	3′06″	1	1 by interviewer 8	1 by interviewee 48 0 by interviewee 49
	Wav.40	22′15″	12	8 by interviewer 8	2 by interviewee 50 2 by interviewee 51
	Wav.41	21′30″	4	3 by interviewer 8	1 by interviewee 52 0 by interviewee 53
	Wav.42	22′01″	5	0 by interviewer 8	5 by interviewee 54 0 by interviewee 55
	Wav.43	8′08″	4	1 by interviewer 8	3 by interviewee 56 0 by interviewee 57
	Wav.44	21′07″	8	5 by interviewer 8	3 by interviewee 58 0 by interviewee 59
	Wav.45	20′53″	9	4 by interviewer 8	3 by interviewee 60 2 by interviewee 61
MING REN MIAN DUI MIAN	Wav.46	23′32″	3	1 by interviewer 9	2 by interviewee 62
	Wav.47	23′09″	6	0 by interviewer 9	6 by interviewee 63
	Wav.48	23′09″	5	0 by interviewer 9	5 by interviewee 64
	Wav.49	23′51″	3	0 by interviewer 9	3 by interviewee 65
	Wav.50	24′44″	4	0 by interviewer 9	4 by interviewee 66
	Wav.51	24′19″	6	0 by interviewer 9	6 by interviewee 67
	Wav.52	24′50″	4	1 by interviewer 9	3 by interviewee 68
	Wav.53	16′31″	5	0 by interviewer 9	5 by interviewee 69
HER VILLAGE	Wav.54	20′34″	7	5 by interviewer 10 0 by interviewer 11	2 by interviewee 70
	Wav.55	18′22″	9	1 by interviewer 10 0 by interviewer 11	5 by interviewee 71
	Wav.56	38′14″	12	4 by interviewer 10 0 by interviewer 11	8 by interviewee 72
	Wav.57	19′28″	6	2 by interviewer 10 0 by interviewer 11	4 by interviewee 73
	Wav.58	27′34″	9	1 by interviewer 10 0 by interviewer 11	3 by interviewee 74 5 by interviewee 75
	Wav.59	18′33″	8	0 by interviewer 10 0 by interviewer 11	8 by interviewee 76
	Wav.60	19′47″	7	4 by interviewer 10 0 by interviewer 11	2 by interviewee 77 1 by interviewee 78
XING YUE DUI HUA	Wav.61	4′52″	2	0 by interviewer 12	2 by interviewee 79
	Wav.62	9′58″	6	0 by interviewer 12	6 by interviewee 80
	Wav.63	4′57″	3	0 by interviewer 12	3 by interviewee 81
	Wav.64	7′35″	7	1 by interviewer 12	6 by interviewee 82
	Wav.65	7′15″	6	0 by interviewer 12	6 by interviewee 83
	Wav.66	4′59″	3	0 by interviewer 12	3 by interviewee 84
	Wav.67	9′45″	5	0 by interviewer 12	5 by interviewee 85
	Wav.68	7′33″	4	0 by interviewer 12	4 by interviewee 86
	Wav.69	8′07″	5	0 by interviewer 12	5 by interviewee 87
	Wav.70	4′57″	3	1 by interviewer 12	2 by interviewee 88
	Wav.71	18′36″	8	0 by interviewer 12	4 by interviewee 89 4 by interviewee 90
Total	71 wave files	32 h 2′47″	490	91 by **10** interviewers	399 by **73** interviewees

From this audio corpus, sentences^2^ embedding 490 discourse marker *ni zhidao* tokens were manually extracted as the subject of research, the corresponding audio of which totals 1 h 30′23″, the related texts were manually transcribed, and the pragmatic and prosodic features were manually annotated and marked up. The annotation and mark-up of pragmatic features was the annotation and mark-up of the functional factors that affect the prosodic characteristics of discourse. It covered three dimensions: turns (the progression of turns and the types of turn-taking), discourse functions (the functions of turn-composing and non-turn-composing linguistic units), and paralinguistic information (mood, attitude, etc.) (Xiong, 2003). In the annotation and mark-up of prosody, Praat speech analysis software was used to characterize prosodic parameters, such as duration, tempo, pause (speech break-off), pitch, and intensity. On this basis, all discourse marker tokens of *ni zhidao* were identified and extracted from the corpus and then classified according to their discourse positions. Subsequently, the discourse functions and prosodic features of all *ni zhidao* tokens were examined in detail.

“The courts, education, police, social services, medicine, business meetings, and mass media have all been major areas of institutional talk research during the past 20 years” (Heritage, 1997: 106–107). The data of this research was selected from one distinct category of institutional talk, interview speech, which is institutional by nature and thus relatively formal. The institutional talk takes place in social institutions of various walks of life. The defining characteristics of institutional talk lie in the fact that the interactions in institutional settings are conducted between laypeople and representatives of public institutions. Thus, the institutional talk features the following attributes: the interaction targets goals tied to relevant identities in institutional organizations; the interaction exerts particular constraints on allowable contributions to the business at hand; the interaction involves special inferences particular to specific contexts (Heritage, 1997: 106). As a result, the activities of the conversationalists tend to be strongly influenced by such restrictions of goal orientation, special constraints on contributions, and special inferences. In this respect, there is little “stylistic” difference between different speakers in the data.

In addition, since all of the conversations from which the data was extracted are media interviews, the participants fall into two groups according to their roles: hosts/interviewers and guests/interviewees. The hosts function as the monitors and controllers of the talk flow. They “despite differences in style are all adept at managing outrage, encouraging the telling of secrets, cooling off the proceedings if they threaten the continuity of the show, shutting off boring guests, putting people on the spot, summing up with clichés and platitudes complex situations making the audience feel comfortable witnessing private matters” (Abt and Seesholtz, 1994: 211). They have to elicit the guests’ account of personal experiences and viewpoints on specific topics by establishing enough rapport with the guests so that discourse is facilitated instead of being hindered. The guests, under the hosts’ elicitation, narrate their personal experiences in a vernacular style due to the stressful situations of interviews and their emotional display. So, the stylistic differences that should be considered are speaker roles (interviewers vs. interviewees) and speakers’ emotional states. Besides, speaking styles caused by the speakers’ physiological variations ought to be considered. These factors will be discussed in section “Results and Discussion.”

Combining computational statistics and modeling, modern speech technology, and discourse analysis, we conducted an empirical study of the actual usage of *ni zhidao*. The specific procedures of the experiments involved the analysis of its prosodic parameters, the investigation of its pragmatic functions, and the examination of the interaction between its prosody and pragmatics.

**FIGURE 1:**
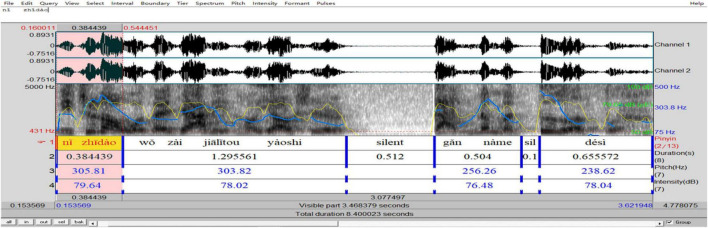
Prosodic features of *ni zhidao* manifested through Praat.

In analyzing the prosody of *ni zhidao*, we drew on Praat speech technology to visualize and label the prosodic features and output results (see Figure 1).

**FIGURE 2:**
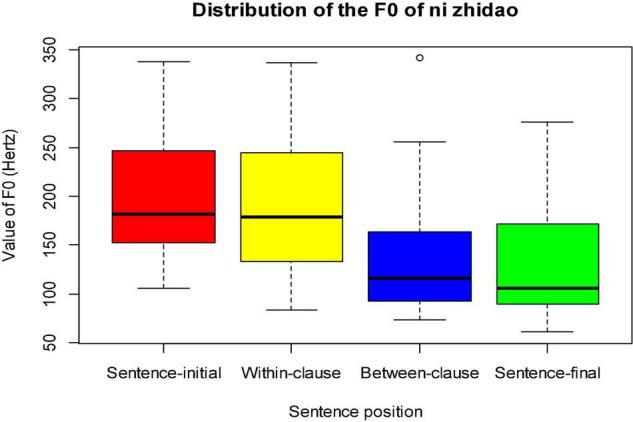
Distribution of the F0 of *ni zhidao* in four sentence positions.

In this process, we first inputted the wave files into Praat and obtained the sound spectrograms. In these spectrograms, we separated *ni zhidao* from the rest of its embedding sentence by acoustically determining its onset and offset^3^. Then we added scripts in Chinese *pinyin* and duration (and pauses, if they did occur) manually. Based on duration, we got the tempo^4^ values. After that, we used Praat “Pitch” and “Intensity” functions (shown at the top of Figure 1) to collect the values of F_0_ and intensity semi-automatically. In these functions, there are sub-functions, including “Pitch listing,” “Get pitch,” “Get minimum pitch,” and “Get maximum pitch,” which can provide the corresponding value (s) of the selected audio part. “Pitch listing” is used to obtain a list of pitches corresponding to any point of time during the duration of *ni zhidao* in time sequence in a new automatically pop-up window; “Get pitch” is used to show the mean pitch of *ni zhidao* during its duration in a new automatically pop-up window; “Get minimum pitch” is used to display the minimum pitch of *ni zhidao* over its duration in a new automatically pop-up window; “Get maximum pitch” is used to get the maximum pitch of *ni zhidao* over its duration in a new automatically pop-up window. In the same vein, the mean, minimum, and maximum intensity of *ni zhidao* can be obtained through “Intensity listing,” “Get intensity,” “Get minimum intensity,” and “Get maximum intensity” functions. Besides, once *ni zhidao* and the pause before and after it is selected, the value of its duration and pre-pause and post-pause will automatically be shown at the bottom panel of the Praat window. All values of the collected prosodic parameters were checked and adjusted by three doctoral students majoring in phonetics and their supervisor. Finally, based on the values obtained, we used the R boxplot to plot the distribution of prosody (see Figure 2).

In the pragmatic investigation of *ni zhidao*, we relied not merely on context, Schiffrin (1987), and Matzen (2004) as the main determinants of pragmatic functions (see Figures 6–8), but on position and prosody as supplementary references as well. Subsequently, we invited four doctors of pragmatics to verify the pragmatic functions inferred.

In terms of the correlation of prosody and pragmatics of *ni zhidao*, we used the random forest in R to construct a pragmatics classification model based on prosody (see Figure 11) to find out the importance of prosody to the performance of pragmatic functions (see Figure 12), and then to use the model constructed to predict pragmatic functions. Subsequently, based on the statistical evidence, we objectively interpreted the interaction between the prosody and pragmatics of *ni zhidao*.

This corpus-based research used prosodic evidence to objectively reveal not merely the role of *ni zhidao* in dynamically constructing and embodying specific contexts but also its communicative functions and the meta-pragmatic awareness underlying it. Such a study can, to a large extent, break through the limitations of traditional discourse marker research, which mainly relies on context and discourse characteristics for subjective and intuitive reasoning. Therefore, the method for discourse marker studies can be made more rigorous, the scope of research can be made more extensive, and the findings can be made sounder.

## Results and Discussion

### Sentence Positions of *ni zhidao*

**FIGURE 3:**
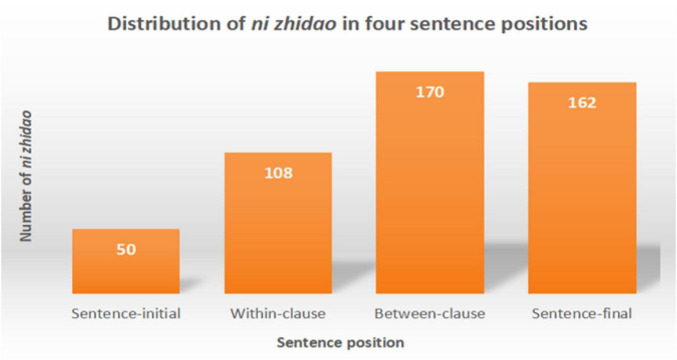
Distribution of *ni zhidao* in four sentence positions.

It was found that the 490 tokens of *ni zhidao* were used in four sentence positions: 50 at the beginning of sentences, 108 within clauses, 170 between clauses^5^, and 162 at the end of sentences. Figure 3 illustrates this distribution.

As Figure 3 shows, most *ni zhidao* tokens were used in the middle (within-clause and between-clause) and at the end of sentences. This discovery indicates that the findings of some studies (e.g., Schiffrin, 1987; Fraser, 1990; Hansen, 1997; Schourup, 1999) need to be updated, which advocate that occurring at the beginning of a sentence (initiality) is one of the defining features of discourse markers.

### Distribution of Prosodic Parameters of *ni zhidao*

**FIGURE 4:**
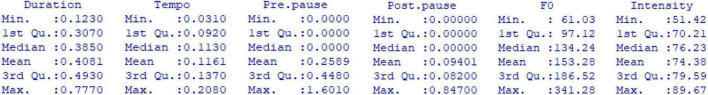
Profile of values of prosodic parameters of *ni zhidao* in four sentence positions.

**FIGURE 5:**
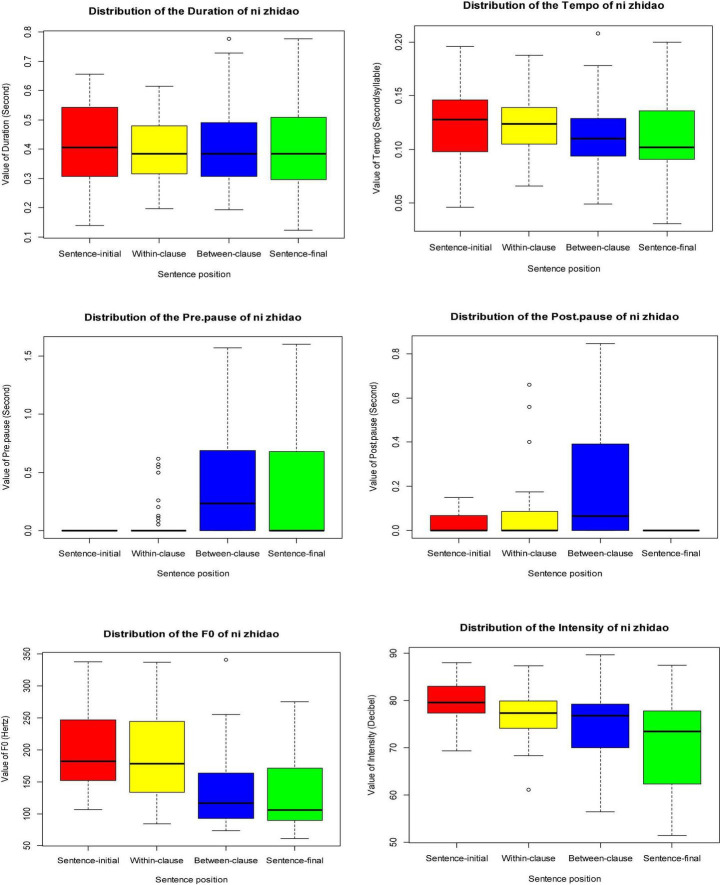
Prosodic features of *ni zhidao* in four sentence positions.

Figure 4 summarizes the values of *ni zhidao*’s prosodic parameters, including their minimum and maximum values, mean values, median values, and 1st and 3rd quartile values, in the dataset as a whole. When occurring in different sentence positions, *ni zhidao* displays different ranges of values regarding duration, tempo, pre-pause, post-pause, F_0_, and intensity, as is illustrated in Figure 5.

Table 3 shows the mean values of *ni zhidao*’s prosodic parameters in four sentence positions.

**TABLE 3:** Mean values of prosodic parameters of *ni zhidao* in four sentence positions.

Prosodic features	Positions			
	Sentence-initial	Within-clause	Between-clause	Sentence-final
Mean pre-pause (second)	—	0.093	0.371	0.328
Mean post-pause (second)	0.194	0.082	0.261	—
Mean duration (second)	0.384	0.372	0.360	0.336
Mean tempo (second/syllable)	0.128	0.124	0.120	0.112
Mean F_0_ (Hz)	196.733	185.053	142.822	131.872
Mean intensity (dB)	79.041	76.920	75.033	70.300

**FIGURE 6:**
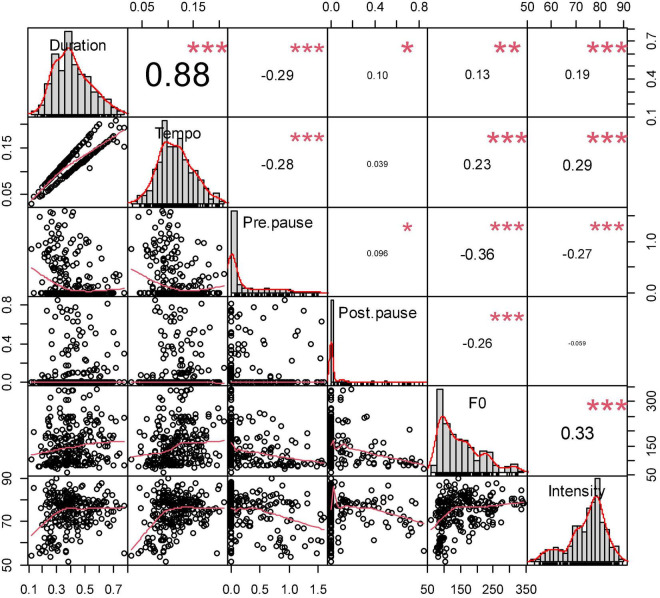
Correlation between the prosodic features of *ni zhidao* in four sentence positions.

Figure 5 and Table 3 show specific correlations between the prosodic parameters and sentence distribution of *ni zhidao*. Clearly, from the sentence-initial to sentence-final positions, the duration of *ni zhidao* shortened gradually, its tempo sped up gradually, and its F_0_ and intensity decreased gradually; sentence-initial *ni zhidao* had the most extended duration, the slowest tempo, the highest F_0_, and the most vigorous intensity. These findings confirm some existing findings: in general, F_0_ is positively correlated with duration (Cai et al., 1998; Xu, 1999; Zhong et al., 2001; Tao, 2001); higher pitch, more vigorous intensity, and longer duration generally focus on the same discourse component (Vaissière, 1983); the discourse component at the beginning of the turn is higher in pitch, which is usually used by the speaker to grab or take over the turn (Braga and Marques, 2004); the speaker draws the listener’s attention to the subsequent discourse by raising the pitch (Ward, 2004); the pitch of the phrase initiating a new topic is higher than that of other components in the same turn (Hirschberg, 2002); intensity increases or decreases automatically as F_0_ increases or decreases (Wu, 2002); the phrase at the end of the sentence has a narrower F_0_ range, lower pitch, and faster tempo (Hirschberg, 2002); there is the effect of final lowering, that is, the phrase at the end of the sentence displays the lowest pitch (Wang, 2011). The package “PerformanceAnalytics” in R was used to demonstrate the correlations between the six prosodic variables of *ni zhidao*, as is illustrated in Figure 6.

The matrix in Figure 6 makes the binary variable correlation well pronounced. The diagonal shows the histograms of the data for the six prosodic variables. The upper right cells (above the diagonal) show the correlation coefficients^6^ for any two variables and significance levels^7^. The lower left cells (below the diagonal) show the scatterplot of any two variables with the fitted lines^8^.

The scatterplot and fitted lines, correlation coefficients, and significance levels all show that there is a significant correlation between any two of the five variables, including duration, tempo, pre-pause, F_0_, and intensity and that there is no significant correlation between post-pause and the four variables of intensity, duration, tempo, and pre-pause, and post-pause is only significantly correlated with F_0_.

As is statistically proved in Figure 6, the F_0_ of *ni zhidao* is positively correlated with its duration, tempo, and intensity, and negatively correlated with its pre-pause and post-pause; the existent studies (e.g., Vaissière, 1983; Cai et al., 1998; Xu, 1999; Zhong et al., 2001; Hirschberg, 2002; Wu, 2002; Ward, 2004; Braga and Marques, 2004; Wang, 2011) confirms the impact of intensity, duration (and tempo), position, and final lowering effect on F_0_. Therefore, both the statistical evidence and the findings in the literature can somehow explain the wide range of the mean values of *ni zhidao* from 61.03 to 341.28 Hz. *Ni zhidao*’s pitch contours of greatly varying ranges revealed in the acoustic experiment, such 75.30–105.17 Hz, 80.91–99.46 Hz, 123.85–192.53 Hz, 146.63–236.91 Hz, 209.16–266.06 Hz, 185.54–277.47 Hz, 182.71–311.54 Hz, 217.89–397.14 Hz, 120.52–407.84 Hz, etc., also justify this wide range to some extent. There is a rich linguistic tradition characterizing variation in overall pitch contour in many different ways: syntactic mood, speaker attitudes, and speaker beliefs (Bolinger, 1989). Some inherent meaning has often been sought in particular contours-often modulated by context (Liberman and Sag, 1974). The expressive content of prosody, including the identity of the speakers, their attitude, mood, age, sex, sociolinguistic group, and other extralinguistic features (d’Alessandro, 2006), is likely to influence the pitch contour produced. Additionally, this wide range may be attributed to the diverse emotional states of the speakers, that is, the correlation of prosodic features with emotional speech (Hirschberg, 2002), typically exemplified by speakers 37, 39, 42, 65, etc., and to their distinct speaking habits caused by diversified physiological attributes. The fact that word boundaries, morphological and phonological word structures, and juncture phenomena may all contribute to determining F_0_ contour (Vaissière, 1983: 63) can be counted as another contributor.

Such associations deepen our understanding of prosody, and thus, to some extent, overcome the subjectivity and limitation of judging prosody merely from context. Besides, this can also provide quantitative and visual clues for investigating the pragmatics of *ni zhidao*.

### Pragmatic Functions of *ni zhidao*

Based on some previous studies (including Schiffrin, 1987; Lenk, 1995, 1998; Aijmer, 1996, 2002; Brinton, 1996; Hansen, 1998; Jucker and Ziv, 1998; Erman, 2001), Shan (2014a) and Shan (2014b) explored the pragmatic functions of *ni zhidao* on the interpersonal plane and the textual plane. On the interpersonal plane, *ni zhidao* constructs cognitive context, projects mental attitudes or checks the hearer’s comprehension/attention, and make relevant inference ostensive from the perspectives of cognition, psychology, and social interaction. Specifically, *ni zhidao* implies not only the speaker’s attitudes, evaluation, judgments, expectations, and demands, but the nature of the social exchange, the role played by the speaker, and the role assigned to the hearer as well. In this way, it indicates the speaker’s intentions, wishes, and emotions, and in the meantime, takes into consideration the hearer’s face, feelings, and social status. On the textual plane, *ni zhidao* helps to ensure the fluent progression of a particular stretch of discourse by creating coherence between the preceding and following sentence segments, by eliciting new topics/turns, and by emphasizing topics. Therefore, *ni zhidao* provides coordinates within the context by indexing sentences either to the texts (textual functions) or to the participants (interpersonal functions) (Schiffrin, 1987: 316–317).

**FIGURE 7:**
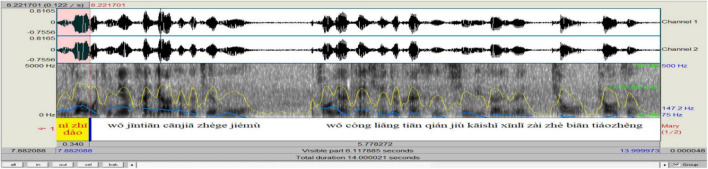
Praat analysis of sentence-initial *ni zhidao*.

**FIGURE 8:**
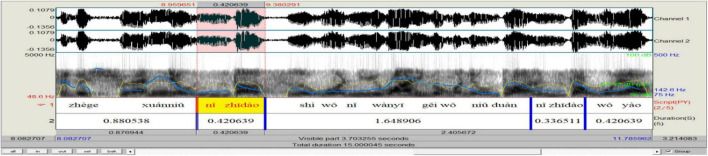
Praat analysis of within-clause *ni zhidao.*

**FIGURE 9:**
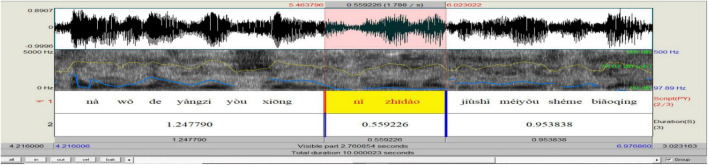
Praat analysis of between-clause *ni zhidao.*

**FIGURE 10:**
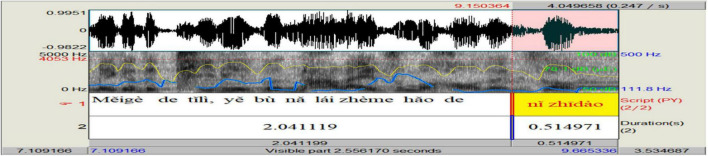
Praat analysis of sentence-final *ni zhidao.*

**FIGURE 11:**
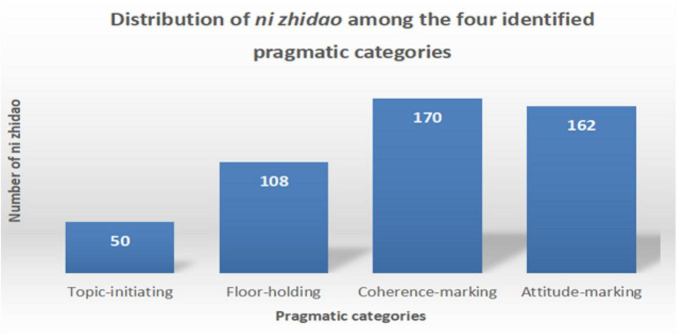
Distribution of *ni zhidao* among the four identified pragmatic categories.

Concerned with the communicative function of *ni zhidao* in simultaneous speech, this study only focuses on its interpersonal functions. Turn position serves to differentiate functional categories (Matzen, 2004). When occurring sentence-initially, -medially (within clauses and between clauses), and -finally, *ni zhidao* displays “subtle changes in meaning or function” (Hansen, 1997: 156). The functional load of discourse markers is embedded in dialog context; humans are better at identifying the communicative functions of discourse markers when they are judged in combination with corresponding dialog sections: “even a very limited context appears to suffice” (Gravano et al., 2007: 804). Drawing on the context in data analysis and based on Schiffrin (1987) and Matzen (2004), the current research found that *ni zhidao* mainly fulfills four categories of pragmatic functions, including that of initiating a topic (by grabbing/taking over the turn) when occurring sentence-initially (see Figure 7), of holding the floor when appearing within clauses (see Figure 8), of marking coherence when making its presence between clauses (see Figure 9), and of projecting attitudes and feelings when showing up sentence-finally (see Figure 10). The distribution of the 490 *ni zhidao* tokens among these four pragmatic categories is illustrated in Figure 11.

It needs to be pointed out that each of these four broad functions can be divided into specific sub-functions. Take the floor-holding function for instance. This umbrella function will likely consist of the sub-functions of stalling time for thinking (lexical/content search), marking the false start, indicating reformulation, etc. These sub-functions will be studied in separate papers devoted to examining the sentence-initial *ni zhidao*, the within-clause *ni zhidao*, the between-clause *ni zhidao*, and the sentence-final *ni zhidao*, respectively. In these prospective examinations, the minute prosody-pragmatics correlations of these sub-functions will be scrutinized, as claimed by Schegloff (1982); Ward and Tsukahara (2000), Gravano and Hirschberg (2009), and Buschmeier et al. (2011). The current study only makes an initial attempt to probe one broad prosody-dependent function at each of the four given sentence positions, that is, the topic-initiating function of the sentence-initial *ni zhidao*, the floor-holding function of the within-clause *ni zhidao*, the coherence-marking function of the between-clause *ni zhidao*, and the emotion-projecting function of the sentence-final *ni zhidao*. These impressionistic pragmatic categories based on immediate context and careful listening can be substantiated by acoustic data (Volín et al., 2016) in the following section.

### Importance of Prosodic Variables of *ni zhidao* to Its Pragmatics and Modeling of Its Prosody-Pragmatics Correlation

Pragmatic functions, such as holding the floor, embodying thought hesitation, managing interpersonal relationships, and expressing emotions and attitudes, rely heavily on prosody (Braga and Marques, 2004). Thus, it can tentatively be argued that the prosodic features of *ni zhidao* jointly reflect the communicative intent of the speaker and the underlying meta-pragmatic awareness involved. The four categories of pragmatic functions fulfilled by *ni zhidao* showed acoustic properties which were sufficiently specific to allow the Random Forest algorithm to distinguish them from each other statistically (Volín et al., 2016).

In this section, the Random Forest^9^ classification model^10^ (see Endnote i for the script) in R is used to test the importance of the prosodic variables of *ni zhidao*, including duration, tempo, pre-pause, post-pause, F_0_, and intensity, to the performance of its pragmatic functions, to build a pragmatics classification model based on prosody, and to use this model to predict the pragmatic functions of *ni zhidao*. The first five rows of the dataset (‘‘Ni zhidao.csv’’^11^) below provide an overview of the research data:

**Table:** 

Position	Duration	Tempo	Pre-pause	Post-pause	F_o_	Intensity	Pragmatics
1 Sentence-initial	0.294	0.098	0	0	200.437	78.449	Topic-initiating
2 Sentence-initial	0.261	0.087	0	0.036	106.316	87.978	Topic-initiating
3 Sentence-initial	0.384	0.128	0	0	305.818	79.646	Topic-initiating
4 Sentence-initial	0.656	0.164	0	0.114	149.297	73.517	Topic-initiating
5 Sentence-initial	0.412	0.137	0	0.108	337.744	79.253	Topic-initiating

**FIGURE 12:**
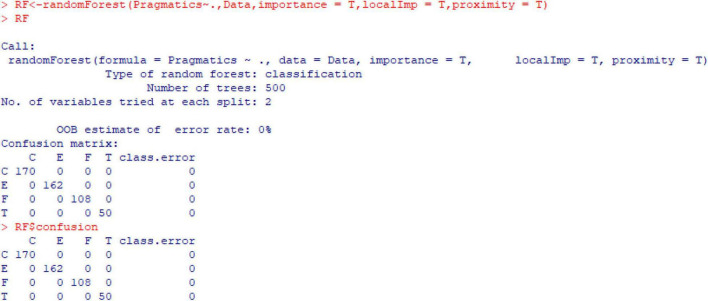
Random forest pragmatics classification model of *ni zhidao* based on its prosody. In this model, “C,” “E,” “F,” and “T” refers to the pragmatic functions of “coherence-marking,” “emotion-projecting,” “floor-holding,” and “topic-initiating” respectively.

During the building of the random forest classification model, “Pragmatics” was set as the dependent variable, and “Duration,” “Tempo,” “Pre-pause,” “Post-pause,” “F_0_,” and “Intensity” were nominated as the independent variables. Figure 12 illustrates the established pragmatics classification model based on prosody.

From Figure 12, it is seen that the “OOB estimate of error rate” is 0%. The confusion matrix indicates that the classification error of the random forest classification model is 0. In other words, this classification model can perfectly explain all the data in the dataset because no function is attributed to the wrong class, as is displayed in the “Confusion matrix,” particularly in the column of “class.error.” This result, therefore, shows that the established model functions effectively at this stage of statistical analysis.

**FIGURE 13:**
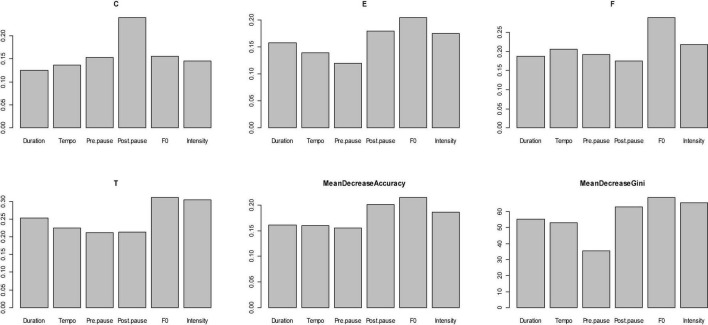
Result of the variable importance in the classification model.

In this model, the importance of the independent variables was statistically tested and visualized through Figure 13. This figure shows that the six prosodic variables (‘‘Duration,’’ ‘‘Tempo,’’ ‘‘Pre-pause,’’ ‘‘Post-pause,’’ ‘‘F_0_,’’ and ‘‘Intensity’’) are all important^12^ to the performance of the four pragmatic functions of “coherence-marking,” “emotion-projecting,” “floor-holding,” and “topic-initiating” respectively and collectively. Specifically, these variables contribute to each of these four functions differently, which is illustrated by the different heights of the individual bars. As is proved by “MeanDecreaseAccuracy” and “MeanDecreaseGini,” “Post-pause,” “F_0_,” and “Intensity” are overall more important than “Duration,” “Tempo,” and “Pre-pause.”

**FIGURE 14:**
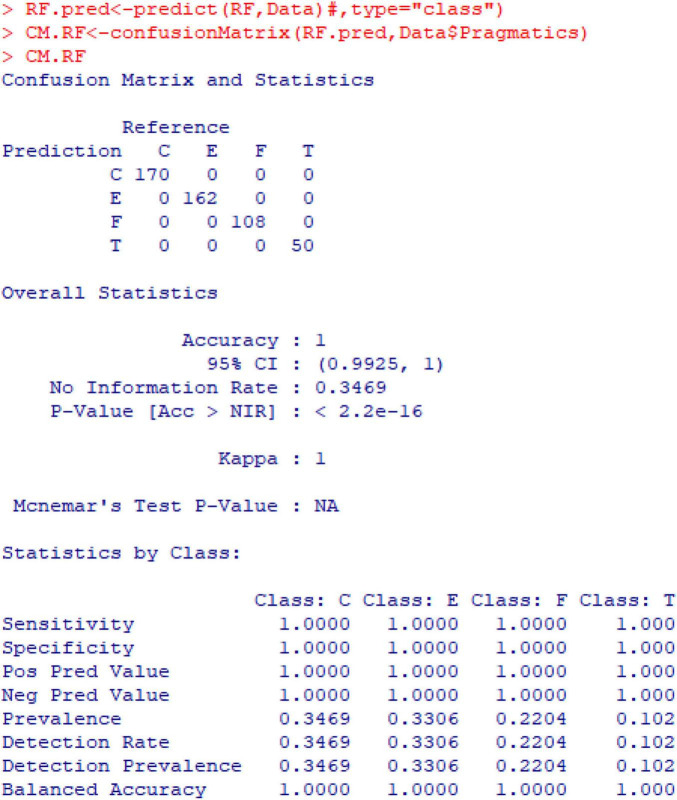
Result of confusion matrix in the prediction of pragmatics.

Given that the classification model established above has been proved effective and the six prosodic variables have all been proved important in the model, this model can be applied to predict the pragmatics of *ni zhidao* (see Figure 14). In this figure, the “Confusion Matrix and Statistics” shows that the “Prediction” perfectly matches the “Reference,” that is, none of the four categories of functions is inaccurately predicted; the “Overall Statistics” indicates that the overall accuracy rate of prediction by the pragmatics classification model based on prosody is 100%; the “Statistics by class” displays that the “Balanced Accuracy” of each class of function is also 100%. This means that the constructed model can perfectly predict the pragmatic functions of *ni zhidao*.

**FIGURE 15:**
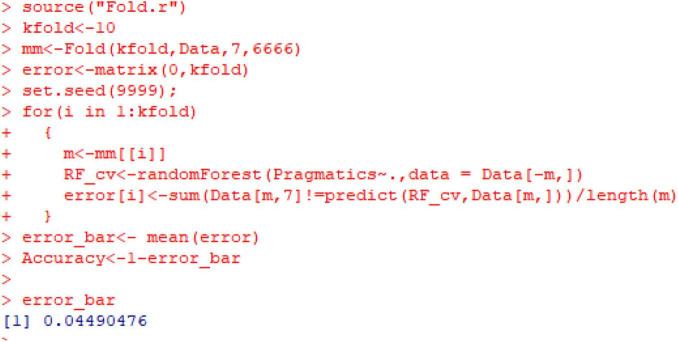
Result of K-fold cross-validation of the performance of the established classification model.

Next, the K-fold cross-validation (see Figure 15) is used to validate the performance of the established classification model. As can be seen, the dataset of the current study is divided into ten sub-datasets, each of which is used as a testing set and the remaining nine of which are used as a training set in a recycling manner. In this way, the error of the 10-fold cross-validation (just below 0.045) is finally worked out. By drawing on all the data in the dataset, this way of validation can most accurately evaluate the performance of the established model when it is used on the test set. The reasonably low error indicator of just below 0.045 shows that the constructed model performs satisfactorily, and thus there is no need to improve the model.

### Discussion

The prosodic features of a sentence are primarily constrained by its communicative functions, and the latter can be realized through the former to some extent (Xiong and Lin, 2004: 116). As the results of the random forest algorithm in section “Importance of Prosodic Variables of *Ni Zhidao* to Its Pragmatics and Modeling of Its Prosody-Pragmatics Correlation” indicate, the six prosodic parameters of *ni zhidao* are significantly related to its pragmatic functions, and thus its pragmatics can objectively be revealed and understood through its prosody.

Sentence-initial *ni zhidao* is designed to initiate a topic. To this end, the speaker mainly resorts to duration, F_0_ and intensity, as is statistically proved in Figure 13. The acoustic experiment showed that sentence-initial *ni zhidao* is characteristic of a contour of a sharp rise followed by an abrupt fall, usually with a reset. Statistics in Table 3 show that sentence-initial *ni zhidao* is uttered with the highest mean F_0_ among *ni zhidao* in the four sentence positions. It was also found that the mean F_0_ of sentence-initial *ni zhidao* is higher than that of other components in the same sentence, which is in tune with the finding in Hirschberg (2002). This high-pitched *ni zhidao* servers the speaker’s communicative purpose of grabbing or taking over the turn (Braga and Marques, 2004). By raising the pitch, the speaker intends to draw the listener’s attention to the subsequent discourse in the new topic to be initiated (Ward, 2004). We believe that the highest mean F_0_ value of sentence-initial *ni zhidao* is also related to the speaker’s evaluation of the importance of the speech content: when the speakers think the speech content is important, they will raise the pitch, and otherwise, they will lower the pitch. The sentence-initial *ni zhidao* exhibits the most prolonged mean duration, the highest mean intensity, and the slowest tempo among the four categories of *ni zhidao*. This is because “intensity seems to share some of the functions of both F_0_ variation and durational variation” (Vaissière, 1983: 62), F_0_ is positively correlated with duration (Cai et al., 1998; Xu, 1999; Zhong et al., 2001), higher pitch, more vigorous intensity, and longer duration usually focus on the same discourse component (Vaissière, 1983), and intensity increases or decreases automatically as F_0_ increases or decreases (Wu, 2002). This relationship between intensity and F_0_ has a physiological basis. They are regulated by the same mechanisms (increase in pulmonary effort and sub-glottal pressure, the tension of the vocal folds, etc.) (Vaissière, 1983). It was found that out of 50 tokens of sentence-initial *ni zhidao*, 32 (64%) are not followed by pauses, and 18 (36%) are followed by pauses. In natural speech, pauses can appear anywhere in the sentence (Goldiscourse markeran-Eisler, 1968) due to three needs: grammar, expression of meaning, and breathing (Guo, 1993). As a prosodic word, *ni zhidao*’s syllables sound closely connected, “prosodically separate” (Wichmann et al., 2010: 40) from the following utterance components, as they are of an F_0_ variation group and carry only one word stress (Wang, 2011). Therefore, the pauses following it do not result from grammatical needs. Sentence-initially, there is no physiological need for pauses for breathing. The pauses following it are thus out of the need for expression. Whether such a pause occurs or not depends on whether the speaker needs to win time to think or not. The pauses of different duration reflect the different thinking time spent by the speaker in organizing the subsequent speech. If the speaker does not encounter expression obstacles, such as hesitation, thinking, etc., he can fluently say what he wants to express, and there will be no pause in the speech flow. These prominent prosodic features of sentence-initial *ni zhidao* not only attract the listener’s attention to the follow-up sentence but also hint to the listener the importance of the subsequent sentence, encoding and conveying the speaker’s communicative intent and thus meta-pragmatic awareness that “I want to call your attention to the topic to be initiated in the following sentence.”

When used as a floor-holding means, within-clause *ni zhidao* is a relatively independent prosodic word acoustically, and it is easily separated from the preceding and following sentence components. Figure 13 reveals that F_0_ and intensity are the most important indicators of the floor-holding *ni zhidao.* In this usage, *ni zhidao* features a continuous flat F_0_ contour, usually with a reset after it, but occasionally displays a flat contour with a reset within it. It is produced with the second highest mean F_0_ and intensity among *ni zhidao* in the four sentence positions. Within the embedding clause, it sometimes carries a lower mean F_0_ and a lower intensity, a lower mean F_0_ but a similar intensity, or a mean F_0_ higher than the preceding components and lower than the succeeding components but a higher mean intensity. This prosodic performance of within-clause *ni zhidao* can be deemed as a signal of mental hesitation, most possibly indicative of the speaker’s struggle in the production of intended speech. In comparison with other components in the same turn, the mean tempo of within-clause *ni zhidao* is sometimes faster and sometimes slower, and it is faster than that of sentence-initial *ni zhidao* but slower than that of between-clause and sentence-final *ni zhidao*. The tempo of within-clause *ni zhidao* depends on the length of thinking time used by the speaker: the longer the thinking time is, the longer its duration is and the slower its tempo is. In natural speech, people often do not first think about the content to be expressed and then utter it fluently. Instead, they think and speak while correcting, explaining, or supplementing what has been said. Therefore, the speaker often pauses in the middle of the sentence or between the syntactic components (Grosjean et al., 1979). To avoid too long a pause and thus an embarrassing temporary communication break-off, and even a loss of turn, speakers often use fillers to indicate their mental or thinking state while maintaining the current turn. Being “behavior-driven” (Xue and Liu, 2008), within-clause *ni zhidao* suggests that the speaker has something to say, fulfilling a floor-holding function. If the time won through uttering *ni zhidao* and through the possible pause preceding it is not long enough for the speaker to pinpoint what is to be produced, there is likely to be a pause following it. Of the 108 tokens of within-clause *ni zhidao*, 12 (11.1%) are preceded by a pause, 10 (9.3%) are followed by a pause, 20 (18.5%) are both preceded and followed by a pause, and 66 (61.1%) are neither preceded nor followed by a pause. This result shows that because “where there is a pause, there is a danger of losing the turn” (Ward, 2004), the speaker endeavors not to pause within the clause for fear of losing the floor or causing embarrassment. These prosodic performances of within-clause *ni zhidao* are closely related to one another (as is shown in Figure 6), and they jointly decipher the speaker’s intended pragmatic meaning that “I want to hold the floor here, so please allow me time to search for the appropriate content or linguistic items.”

As with the between-clause *ni zhidao*, post-pause is the most important indicator of its coherence-marking function. Of the 170 tokens of between-clause *ni zhidao*, 92 (54.1%) are followed by a pause. This post-pause is most likely to result from the purpose of expression, in that there is no need of pause for breathing after such a short prosodic word, and there is no need of pause for marking the boundary of the prosodically independent *ni zhidao.* This *ni zhidao* serves as a link, which indexes both the preceding and succeeding clauses and thus binds them into a coherent proposition. The speaker employs it, coupled with the pause following it, provides the listener with sufficient time to work out the logic between the clause preceding it and the clause to be uttered. According to statistics, 106 (63.4%) tokens of between-clause *ni zhidao* are preceded by a pre-pause, which may be caused by a grammatical need of marking the clause boundary, by a breathing need following a clause, or by an expressive need for time to prepare the succeeding clause and prepare the listener for it. Thus, this pre-pause is most elusive. The between-clause *ni zhidao* is characteristic of a continuous flat contour. Its mean F_0_ and intensity are relatively lower than sentence-initial and within-clause *ni zhidao* but comparatively higher than sentence-final *ni zhidao*. Its mean tempo is faster than that of sentence-initial and within-clause *ni zhidao* but slower than that of sentence-final *ni zhidao.* Compared with other components in the same sentence, it is sometimes faster and sometimes slower, which is decided by the length of time used by the speaker to prepare the listener for the following clause. In a word, the characteristic prosody of between-clause *ni zhidao* is intended to communicate the speaker’s pragmatic purpose that “I want to make ostensive the coherent semantic relations between the preceding and succeeding clauses.”

The prosody of within-clause and between-clause *ni zhidao* does not show any apparent regularity, and the reasons behind this deserve exclusive studies in the future. However, this research found that like the prosody of sentence-initial *ni zhidao*, the prosody of sentence-final *ni zhidao* also exhibits apparent regularity. As is indicated in Figure 13, F_0_ and intensity are once again the most essential variables facilitating sentence-final *ni zhidao* performing the emotion-projecting function. According to statistics, it has the lowest mean F_0_ and intensity and the fastest mean tempo among the four categories of *ni zhidao*. Compared with other components in the same sentence, its mean F_0_ and intensity are lower, its mean tempo is faster, and its mean duration is shorter. Its F_0_ contour is generally flat, with a moderate rise and, or fall within it. These prosodic attributes have a lot to do with the sentence-final position. The phrase at the end of the topic has a narrower tonal range, a lower pitch, and a faster tempo (Hirschberg, 2002). In general, the sentence-final prosodic word carries the lowest F_0_ in a complete sentence (Wang, 2011). This is the FINAL LOWERING, compression of the pitch range during the last half-second or so of an utterance (Caspers, 1998). The compression of pitch range within about half a second before the end of the speech can indicate that the speaker has finished the current turn (Hirschberg, 2002). 73 (45.1%) of the 162 sentence-final tokens are preceded by a pause, which may be triggered by the speaker’s breathing need and, or grammatical need. These prosodic hallmarks of sentence-final *ni zhidao* convey the speaker’s meta-pragmatic awareness that “I want you to identify with me in terms of the strong attitude and feeling that I have projected into the preceding sentence.”

Prosody is a prominent phonetic feature of natural speech, which is extremely important but often overlooked in the research of spontaneous speech. This characteristic of speech reflects the actual usage of language in the improvised context. It intuitively displays the rhythm of the speaker’s speech, as well as the speaker’s attitude toward and emotion at people and things. It thus provides objective evidence for judging the pragmatic functions of discourse. Praat speech software and algorithms in R can represent the prosodic features of *ni zhidao* and the potential interaction between its prosodic features and pragmatic functions statistically, graphically, and thus visually, therefore achieving relatively simple, effective, and reliable semi-automated processing of spontaneous speech. Accordingly, the “invisible” meta-pragmatic awareness underlying discourse is made “visible” to a large extent. Statistical analysis and modeling can put the study of discourse markers on a more objective and scientific footing.

## Conclusion

The intrinsic mechanism of spoken discourse is maintained by three integral components, phonological (the phonetic and prosodic feature), morphological (the morphological and syntactic configuration), and propositional (the semantic and pragmatic meaning). However, academic circles have mostly been inferring and summarizing the pragmatic functions of discourse markers subjectively in specific contexts, based on a few examples or a small-sized corpus. Although some researchers (e.g., Hirschberg, 2002; Matzen, 2004; Braga and Marques, 2004; Wichmann et al., 2010; Beňuš, 2012; Abuczki, 2014; Cabarrão et al., 2015; Gonen et al., 2015; Volín et al., 2016) have investigated how discourse markers’ prosody relates to their pragmatics, they have neither investigated their prosody-pragmatics interaction statistically nor built a model to predict their pragmatic functions automatically.

Based on a relatively large-scaled corpus of media interviews, this study examined the prosody-pragmatics interaction of *ni zhidao.* It was found that *ni zhidao* exhibited different prosodic features when occurring in four sentence positions, that it fulfilled four broad pragmatic functions of initiating a topic, holding the floor, marking coherence, and projecting emotions, and that the prosody-pragmatics correlation of *ni zhidao* can statistically be investigated and modeled, and the constructed classification model can be used to predict the pragmatics of *ni zhidao* highly accurately. These findings seem to confirm that the prosody of *ni zhidao* plays a role in deciphering its different pragmatic functions.

Speech analyses and computational statistics based on the natural spoken language are well established in computational linguistics and speech engineering. Although not used in the studies of discourse markers, these methods are fully applicable to discourse marker research, proved in this study. Within the framework of this paper, we can not only explain abstract pragmatic phenomena in discourse analysis but also clearly and objectively grasp the intrinsic mechanisms of prosody-pragmatics interaction. A combination of formal and functional approaches and corpus data and intellectual inquiry is not only feasible and effective but also inevitable and potent in the future research of language in general and discourse markers in particular.

## Data Availability Statement

The original contributions presented in the study are included in the article/Supplementary Material, and further inquiries can be directed to the corresponding author/s.

## Author Contributions

The author confirms being the sole contributor of this work and has approved it for publication.

## Conflict of Interest

The author declares that the research was conducted in the absence of any commercial or financial relationships that could be construed as a potential conflict of interest.

## Publisher’s Note

All claims expressed in this article are solely those of the authors and do not necessarily represent those of their affiliated organizations, or those of the publisher, the editors and the reviewers. Any product that may be evaluated in this article, or claim that may be made by its manufacturer, is not guaranteed or endorsed by the publisher.

## Footnotes

1 All the interviews in the form of videos were recorded in the studios of the interviewing programs, where the recording levels were set and controlled by the professionals in charges of video recording. I downloaded from the official websites of the interviewing programs the videos, from which Cool edit Pro 2.1 was used to extract and save as waveform files the fractions of audios containing the DM *ni zhidao*. And then Praat 5304 was employed to analyze the extracted fractions of audios. It can be seen in all of the videos that all speakers used microphones which were pinned on their clothes about 20 centimeters below their mouth, which was maybe stipulated by the working professionals in the studio. Therefore, the difference in the recording settings (including distance to microphone and recording levels)can be overlooked in this study.

2 In this research, merely the 490 *ni zhidao* tokens and their embedding sentences were segmented from the audios for analysis. When deciding the boundaries of the sentences embedding *ni zhidao*, we took into full consideration the context, the meaning of the sentences preceding and following the sentences containing *ni zhidao*, and the indexical functions of *ni zhidao*. The indexical functions of *ni zhidao* provide textual coordinates, which focus on “prior text vs. upcoming text: markers index their containing utterances to whatever text precedes them (proximal), or to whatever text is to follow (distal), or to both” (Schiffrin, 1987: 323). All the sentences containing *ni zhidao* segmented for analysis have been checked and adjusted by five doctors majoring in syntax and five doctors majoring in pragmatics, who consulted the original audios for reference during checking and adjustment.

3 As with the determination of the onset and offset of *ni zhidao*, we drew on acoustic recognition, as well as the characteristics of the sound spectrogram, to determine the boundary of phonemes and/or syllables.

4 There are, generally, two ways of measuring tempo: calculating the mean duration of syllables contained in an sentence, or calculating the number of syllables uttered in a second (Cao, 2003; Li, 2006). Considering that Chinese is a “syllable-timed” (Keller and Zellner, 1996) language, the present study adopts the first approach.

5 In this paper, “clause” merely refers to that within the same sentence where each token of *ni zhidao* is embedded. So, between-clause tokens only refer to those inserted between two clauses embedded in the same sentences. Sentence-initial tokens and sentence-final tokens should not be regarded as between-clause, for the former are merely pragmatically indexed to the clause(s) following them, and the latter are only pragmatically indexed to the clause(s) preceding them.

6 Correlation coefficient: A positive correlation coefficient indicates a positive relationship between variables; a negative correlation coefficient indicates a negative relationship between variables; the closer the data is to 1 or −1, the greater the correlation between variables is.

7 Level of significance: the higher the number of asterisks * is, the more significant the correlation between variables is. The absence of an asterisk * indicates a weak correlation.

8 Fitted line: The closer the distribution is to the fitted line, the more significant the correlation between two variables.

9 The GAMM, the multinomial regression, and the random forest were used as algorithms for categorization, to construct the pragmatics classification model, and the random forest was eventually adopted because it provided the best result of classification.

10 #The script for random forest:

NB: “Fold.r” in this script is a function defined by the authors to divide the dataset randomly. It is designed to ensure the balance between the 10 sub-datasets of data during the K-fold cross-validation.

11 The dataset “Ni zhidao.csv” and the script used for this research have been uploaded to GitHub at https://github.com/victorsyhz/Raw-Data-script for reference.

12 The higher the values are, the more important the variables are.
