# Supplementary material for: Investigating the Interaction Between Prosody and Pragmatics Quantitatively: A Case Study of the Chinese Discourse Marker ni zhidao (“You Know”)
Source: Front Psychol. 2021 Dec 6;12:716791. doi: 10.3389/fpsyg.2021.716791 (PMC8687356; doi:10.3389/fpsyg.2021.716791)
Supplement: Supplementary file 2 [file Data_Sheet_2.docx]

The script:

require(foreign)

require(ggplot2)

require(MASS)

require(Hmisc)

require(reshape2)

##import the file, and named as "dat"

m1 <- polr(Pragmatics~duration+tempo+pre.pause+post.pause+F0+intensity, data=dat)

summary(m1)

#Re-fitting to get Hessian

#Call:

#polr(formula = Pragmatics ~ duration + tempo + pre.pause + post.pause +

# F0 + intensity, data = dat)

#Coefficients:

# Value Std. Error t value

#duration 12.075773 0.949491 12.718

#tempo -37.501378 0.228973 -163.781

#pre.pause 1.219169 0.346366 3.520

#post.pause -2.419429 0.487663 -4.961

#F0 -0.008329 0.002112 -3.945

#intensity -0.078638 0.018624 -4.223

#Intercepts:

# Value Std. Error t value

#FQ|KZ -9.4695 1.4666 -6.4568

#KZ|LG -7.6711 1.4362 -5.3414

#LG|QT -5.5603 1.3963 -3.9822

#Residual Deviance: 523.5096

#AIC: 541.5096

ctable <- coef(summary(m1))

p <- pnorm(abs(ctable[, "t value"]), lower.tail = FALSE) * 2

ctable <- cbind(ctable, "p value" = p)

ctable

ctable

# Value Std. Error t value p value

#duration 12.075773028 0.949491436 12.718148 4.688382e-37

#tempo -37.501378296 0.228972703 -163.781000 0.000000e+00

#pre.pause 1.219168622 0.346366173 3.519884 4.317362e-04

#post.pause -2.419429012 0.487663209 -4.961270 7.003364e-07

#F0 -0.008329408 0.002111536 -3.944716 7.989467e-05

#intensity -0.078638448 0.018623530 -4.222532 2.415734e-05

#FQ|KZ -9.469532588 1.466602494 -6.456782 1.069528e-10

#KZ|LG -7.671102213 1.436154669 -5.341418 9.222240e-08

#LG|QT -5.560265494 1.396285446 -3.982184 6.828490e-05

library(car)

library(splines)

#library(

Anova(m1)

#Analysis of Deviance Table (Type II tests)

#Response: Pragmatics

# LR Chisq Df Pr(Chisq)

#duration 30.205 1 3.888e-08 ***

#tempo 19.693 1 9.094e-06 ***

#pre.pause 12.730 1 0.0003599 ***

#post.pause 25.970 1 3.468e-07 ***

#F0 15.762 1 7.183e-05 ***

#intensity 18.970 1 1.328e-05 ***

#---

#Signif. codes: 0 ‘***’ 0.001 ‘**’ 0.01 ‘*’ 0.05 ‘.’ 0.1 ‘ ’ 1

##all variables are significant

###plot the effects

library(effects)

effects2 <- allEffects(m5, partial.residuals=T)

pdf("effects3.pdf", wi=10, he=8.5)

#plot(effects)

plot(effects1, multiline=TRUE, ci.style="bands", colors = c("red",

"blue", "green"), lwd=2, cex=0.1, smooth.residuals=F, ask=FALSE, lines=list(multiline=TRUE), grid=TRUE)

dev.off()

### import chinese

dd <- read.csv("zhidao.csv", header=T, stringsAsFactors=FALSE, fileEncoding="utf-8")
